# VU0155069 inhibits inflammasome activation independent of phospholipase D1 activity

**DOI:** 10.1038/s41598-019-50806-9

**Published:** 2019-10-04

**Authors:** Sung Kyun Lee, Ye Seon Kim, Geon Ho Bae, Ha Young Lee, Yoe-Sik Bae

**Affiliations:** 1 Department of Biological Sciences, Sungkyunkwan University, Suwon, 16419 Republic of Korea; 2 Department of Health Sciences and Technology, SAIHST, Sungkyunkwan University, Seoul, 06351 Republic of Korea; 3 Present Address: Institute for Stem Cell & Regenerative Medicine Research of Albert Einstein College of Medicine, Bronx, NY 10461 USA

**Keywords:** Inflammasome, Sepsis, Molecular medicine

## Abstract

The inflammasome is a specialized multiprotein oligomer that regulates IL-1β production. Although regulation of the inflammasome is related to crucial inflammatory disorders such as sepsis, pharmacological inhibitors that effectively inhibit inflammasome activity are limited. Here, we evaluated the effects of a phospholipase D1 (PLD1)-selective inhibitor (VU0155069) against sepsis and inflammasome activation. VU0155069 strongly enhances survival rate in cecal ligation and puncture (CLP)-induced sepsis by inhibiting lung inflammation, leukocyte apoptosis, and the production of proinflammatory cytokines, especially IL-1β. VU0155069 also significantly blocked IL-1β production, caspase-1 activation, and pyroptosis caused by several inflammasome-activating signals in the bone marrow-derived macrophages (BMDMs). However, VU0155069 did not affect LPS-induced activation of signaling molecules such as MAPK, Akt, NF-κB, and NLRP3 expression in the BMDMs. VU0155069 also failed to affect mitochondrial ROS generation and calcium increase caused by nigericin or ATP, and subsequent ASC oligomerization caused by several inflammasome-activating signals. VU0155069 indirectly inhibited caspase-1 activity caused by LPS + nigericin in BMDMs independent of PLD1 activity. We demonstrated that a PLD1 inhibitor, VU0155069, shows anti-septic activity as well as inflammasome-inhibiting effects. Our results suggest that VU0155069 can be considered a novel inflammasome inhibitor.

## Introduction

Inflammatory cytokines such as IL-1β, TNF-α, and IL-6 play crucial roles to mediate the pathological progression of diverse infectious or inflammatory diseases^1^. These inflammatory cytokines are produced by several immune cells and inflammatory cells, and contribute to further recruitment of inflammatory cells into injured or event areas leading to tissue and organ damage^2^. Among these pro-inflammatory cytokines, IL-1β and IL-18 production is regulated by the activation of a specialized multiprotein oligomer, the inflammasome^3^. Activation of TLRs by pathogen-associated molecular pattern molecules or damage-associated molecular pattern molecules induces the expression of pro-IL-1β or pro-IL-18, which is processed by active caspase-1 to produce active IL-1β or IL-18^4^. Activation of caspase-1 is mediated by the inflammasome complex which is comprised of the nucleotide-binding domain leucine-rich repeat containing (NLR) protein, the apoptosis-associated speck-like protein containing CARD (ASC) adaptor protein, and pro-caspase-1^5^. Different NLR proteins recognize signal 2 for the cell activation; NLRP3 detects K^+^ efflux, reactive oxygen species (ROS), or lysosomal damage induced by ATP, crystals or nigericin, AIM2 detects dsDNA, and NLRC4 detects flagellin^6^. Although the crucial components of the inflammasome are well known, detailed molecular mechanisms involved in the activation and regulation of the inflammasome are unclear.

Since the inflammasome is closely associated with diverse disorders, it is regarded as an important drug target^7^. Different inflammasome inhibitors have been reported to regulate inflammasome activity^8^. Two small molecules, MCC950 and BHB, inhibit the NLRP3 inflammasome by blocking ASC oligomerization^9^. Some autophagy inducers such as resveratrol and the CB2R agonist also show inhibitory effects against the NLRP3 inflammasome^10^. However, additional pharmacological inhibitors that effectively inhibit inflammasome activities are necessary for effective use of these molecules.

Phospholipase D (PLD) is an enzyme that hydrolyzes phosphatidylcholine into phosphatidic acid (PA) and choline^11^. Generated PA can regulate diverse signaling molecules including kinases, phosphatases and nucleotide binding proteins^12^. PLD involves the regulation of pathological progress of diverse diseases such as infection, cancer and some neurodegenerative disorders^13,14^. Although PLD, especially PLD2 has been known to mediate inflammation during bacterial infection^14^, it remains to be elucidated what is the role of PLD1 in inflammation and regulation of inflammasome. Previously, PLD isoform-selective inhibitors including a PLD1-selective inhibitor (VU0155069) were developed by Scott and colleagues^15^, which facilitates to analyze physiological and pathological function of PLD. In this study, we investigated the effects of a PLD1-selective inhibitor, VU0155069 on the regulation of inflammation and inflammasome.

## Results

### VU0155069 shows therapeutic effects against experimental polymicrobial sepsis

**Figure 1:**
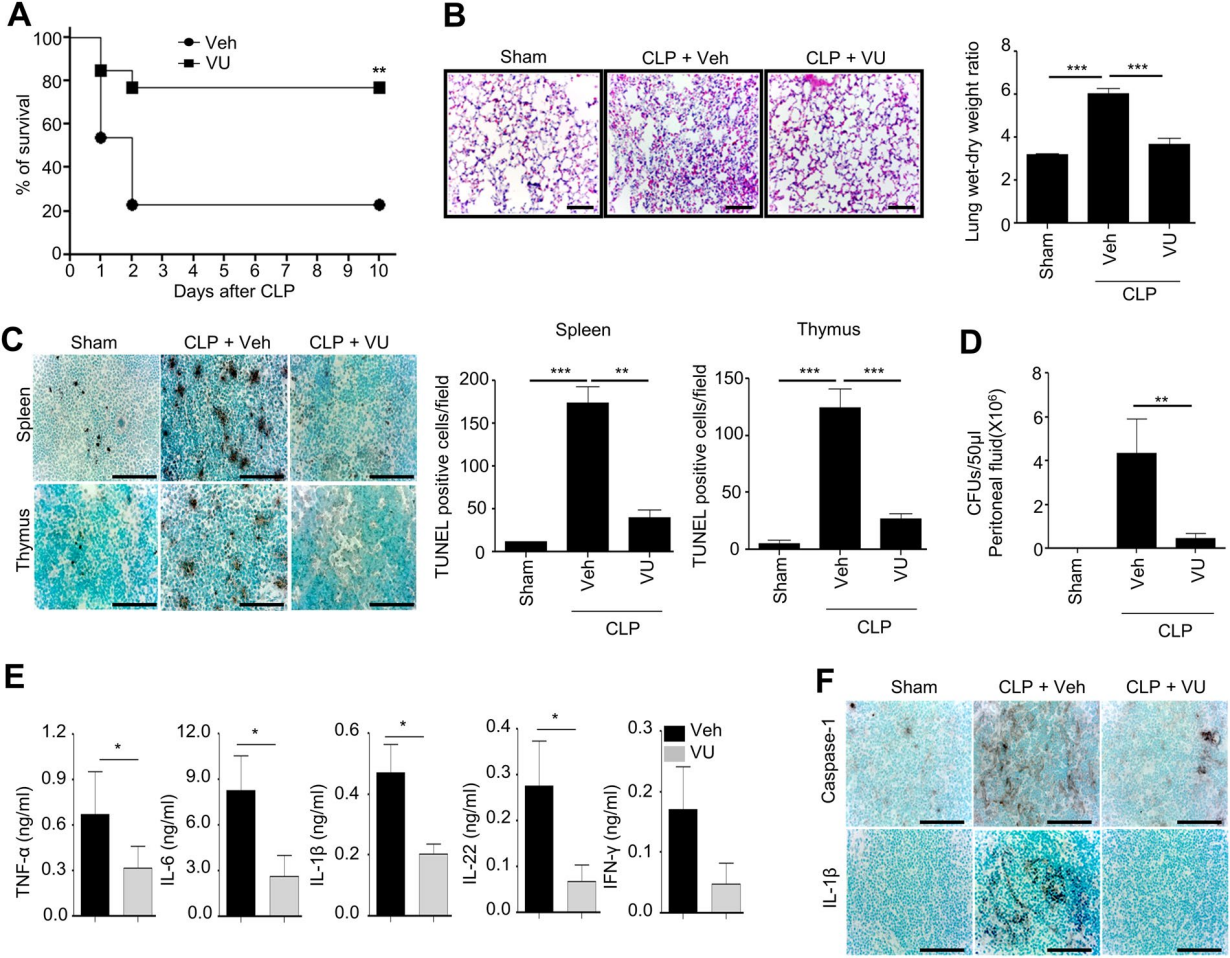
VU0155069 shows therapeutic effects against experimental polymicrobial sepsis. (**A**) C57BL/6 mice were subjected to CLP surgery. Vehicle (0.5% Tween 80 in PBS) or VU0155069 (10 mg/kg) was injected s.c. four times into mice 2, 14, 26, and 38 h after CLP. Survival was monitored for 10 days. **p < 0.01 by log-rank test. Sample size: n = 13 per group (**A**). (**B**–**F**) CLP mice were sacrificed 24 h after surgery. The lungs were stained with hematoxylin and eosin (magnification, ×200) (**B** left). Lungs were used to measure the wet/dry weight ratio 24 h after CLP (**B** right). The TUNEL assay was performed in the spleen and thymus from CLP mice (**C** left), and TUNEL-positive cells were quantified (**C** right). CFU were counted in peritoneal fluid from CLP mice (**D**). Inflammatory cytokines were measured in peritoneal fluid from CLP mice (**E**). Caspase-1 and IL-1β were detected in the spleen of CLP mice using immunohistochemistry (**F**). The data are representative of 3–5 mice per group from two independent experiments. Scale bar, 100 μm (**B** left, **C** left and **F**). Mean ± SEM (n = 3–5 for **B** right, **C** right, **D**,**E**). *p < 0.05, **p < 0.01, ***p < 0.001 by unpaired two-tailed *t*-test (**E**) or by one-way ANOVA followed by Tukey’s multiple-comparisons test (**B** right, **C** right and **D**).

We investigated the effects of a PLD1 inhibitor (VU0155069) against a mouse model of sepsis. Administration of VU0155069 significantly increased survival rate in an experimental animal sepsis model, showing 77% survival comparing to 23% survival for the vehicle control group (Fig. 1A). Polymicrobial sepsis-induced mortality is associated with lung inflammation^14^. We also found that cecal ligation and puncture (CLP) surgery caused marked lung inflammation, showing increased lung congestion and thrombus formation. VU0155069 administration strongly blocked this lung inflammation (Fig. 1B left). Lung wet-to-dry ratio was also increased by CLP, indicating that lung edema is generated, which was significantly decreased by VU0155069 (Fig. 1B right). Previously, widespread leukocyte apoptosis was known to be induced during the pathological progression of sepsis^16^. In our experiments, we also found that CLP operation induced cell death in the spleen and thymus. Administration of VU0155069 significantly decreased TUNEL-positive cells from the spleen and thymus (Fig. 1C). It has been known that septic mortality positively correlates with bacterial burdens^17^. Since we found that administration of VU0155069 significantly increased survival rate in CLP mice (Fig. 1A), we examined the effects of VU0155069 on bacterial burden. Administration of VU0155069 significantly decreased bacterial colony forming units in CLP mice (Fig. 1D).

During the pathological progression of sepsis, a cytokine storm is induced^18^. Measurement of the levels of inflammatory cytokines from the peritoneum showed that levels of several inflammatory cytokines (TNF-α, IL-1β, IL-6, and IL-22) were significantly decreased by VU0155069 administration in CLP mice (Fig. 1E). Among these inflammatory cytokines, IL-1β has been reported to play a crucial role in sepsis pathology^16^. Moreover, IL-1β is known to be processed by active caspase-1 in inflammatory conditions^4^. Immunohistochemical analysis showed that CLP operation elicited marked increase of caspase-1 and IL-1β from the spleen. Administration of VU0155069 almost completely decreased CLP-induced caspase-1 level and subsequent IL-1β level in the spleen (Fig. 1F).

### VU0155069 markedly inhibits inflammasome activation in BMDMs

**Figure 2:**
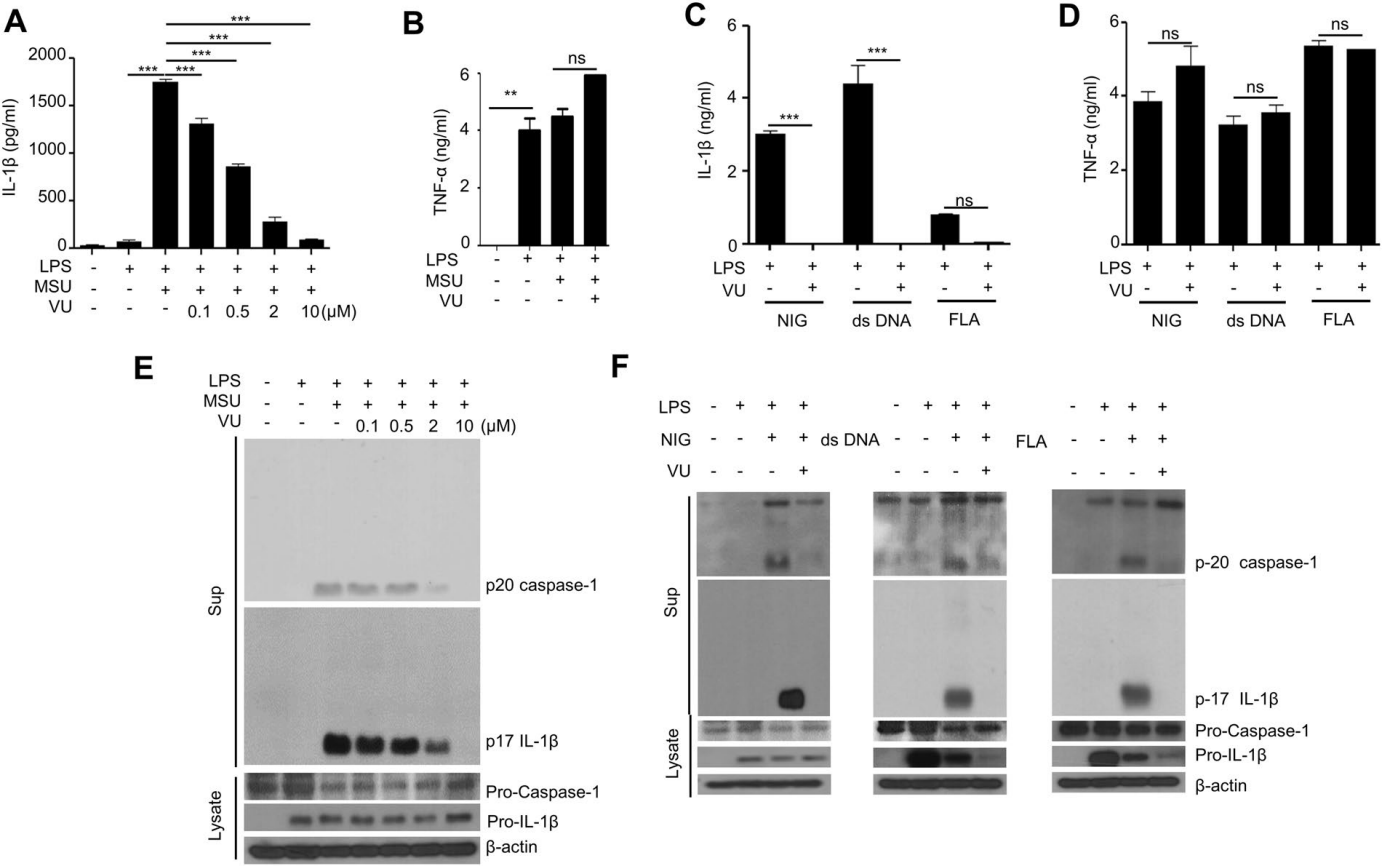
VU0155069 inhibits inflammasome activation in BMDMs. (**A**,**B**,**E**) Generated BMDMs were stimulated with LPS (1 μg/ml) + monosodium urate (MSU) (300 μg/ml)  in the absence or presence of several different concentrations of VU0155069 (0, 0.1, 0.5, 2, 10 μM for A and E, 10 μM for **B–D** and **F**). The levels of IL-1β (**A**) and TNF-α (**B**) were measured by ELISA. (**C**,**D**,**F**) BMDMs were stimulated with LPS (1 μg/ml) for 4 h prior to adding nigericin (NIG) (10 μM) for 30 min, dsDNA (1 μg/ml) or flagellin (FLA) (1 μg/ml) for 16 h in the presence of vehicle (0.05% DMSO) or VU0155069 (10 μM). The levels of IL-1β (**C**) and TNF-α (**D**) were measured by ELISA. Immunoblot of IL-1β and caspase-1 from supernatant or cell lysate (**E,F**). Full blots in Supplementary Fig. S1. Mean ± SEM (n = 3 for **A–D**). **p < 0.01, ***p < 0.001 by one-way ANOVA followed by Tukey’s multiple-comparisons test. The data are representative of three independent experiments (**E,F**). ns, not significant.

Since we found that VU0155069 administration blocks IL-1β production from the spleen in CLP mice (Fig. 1E) and inflammasome activation is apparent in macrophages, we examined the effects of the PLD1 inhibitor VU0155069 on inflammasome activation in macrophages. Stimulation of mouse BMDMs with LPS + monosodium urate (MSU) strongly induced IL-1β production. Addition of VU0155069 significantly inhibited IL-1β production induced by LPS + MSU in a concentration-dependent manner, showing almost complete inhibition at 10 μM (Fig. 2A). Unlike IL-1β, addition of VU0155069 did not affect LPS-stimulated TNF-α production in BMDMs (Fig. 2B). Next, we tested if VU0155069 blocks inflammasome activation caused by different stimuli such as LPS + nigericin, LPS + dsDNA, or LPS + flagellin which activates NLRP3, AIM2 or NLRC4 inflammasome respectively^6^. VU0155069 almost completely blocked IL-1β production induced by these stimuli (LPS + nigericin, LPS + dsDNA, or LPS + flagellin) (Fig. 2C), while TNF-α production was not affected (Fig. 2D). The results indicate that VU0155069 selectively blocks IL-1β but not TNF-α production in BMDMs.

IL-1β production is accompanied by the activation of caspase-1 in BMDMs^5^. Stimulation of BMDMs with LPS + MSU generated processed caspase-1 (p20 caspase, an active form of caspase-1), and processed active p17 IL-1β (Fig. 2E). However, addition of VU0155069 blocked the activation of caspase-1 and IL-1β in a concentration-dependent manner, showing maximal activity at 10 μM (Fig. 2E). Other inflammasome-activating stimuli including LPS + nigericin, LPS + dsDNA, and LPS + flagellin also strongly elicited the processing and subsequent generation of active caspase-1 (p20) and active IL-1β (p17), which was blocked by VU0155069 addition (Fig. 2F).

**Figure 3:**
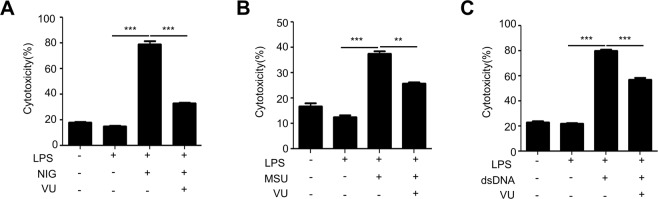
VU0155069 prevents pyroptosis in BMDMs. (**A–C**) BMDMs were stimulated with LPS (1 μg/ml) for 4 h prior to adding nigericin (NIG) (10 μM) for 30 min, monosodium urate (MSU) (300 μg/ml) or dsDNA (1 μg/ml) for 16 h in the presence of vehicle (0.05% DMSO) or VU0155069 (10 μM). LDH release was measured by using a commercial LDH assay kit. Mean ± SEM (n = 4 for **A**–**C**). **p < 0.01, ***p < 0.001 by one-way ANOVA followed by Tukey’s multiple-comparisons test.

Production of the inflammatory cytokine IL-1β was previously associated with pyroptosis, a specialized cell death that is mediated by activation of caspase-1^19^. We also observed that stimulation of BMDMs with inflammasome-activating stimuli (LPS + nigericin, LPS + MSU, LPS + dsDNA) increased cell death. Addition of VU0155069 significantly decreased cytotoxicity caused by the three different inflammasome-activating stimuli (Fig. 3). The results are consistent with our findings that VU0155069 significantly blocks inflammasome activation and subsequent IL-1β production caused by LPS + nigericin, LPS + MSU, or LPS + dsDNA.

### VU0155069 does not affect LPS-stimulated signal 1, ASC oligomerization or membrane pore formation

**Figure 4:**
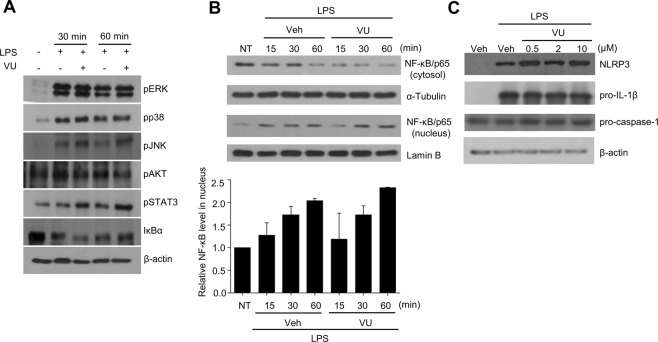
VU0155069 does not affect LPS-stimulated signal 1. (**A,B**) BMDMs were stimulated with LPS (1 μg/ml) in the absence or presence of VU0155069 (10 μM) for 15, 30 or 60 min. (**C**) BMDMs were stimulated with LPS (1 μg/ml) in the absence or presence of VU0155069 (10 μM) for 4 h. The levels of pERK, pp38, pJNK, pAkt, pSTAT3, IκBα, β-actin (**A**), NLRP3, pro-IL-1β, pro-caspase-1, β-actin (**C**) were measured from whole cell lysates with Western blot analysis. Harvested cells were subjected to separation of cytosolic and nuclear fractions, and the levels of NF-κB/65, α-tubulin, lamin B were measured from the cytosol or nuclear fraction with Western blot analysis (**B** top). Relative NF-*k*B levels in the nucleus were quantified using imageJ (**B** bottom). Full blots in Supplementary Fig. S2. The data are representative of three independent experiments (**A,B** top, **C**). Mean ± SEM (n = 3 for **B** bottom).

For proper activation of the NLRP3 inflammasome and subsequent IL-1β production in macrophages, at least two different signals are required; signal 1 is induced by LPS to stimulate the signaling cascade including activation of MAPK and transcription factors such as NF-κB, and signal 2 is induced by particulates such as alum, uric acid crystals, etc. to induce lysosome damage, or by ATP to induce K^+^ efflux^20^. Our finding that VU0155069 blocks NLRP3 inflammasome activation in BMDMs led us to test if VU0155069 affects signal 1 induced by LPS. Stimulation of BMDMs with LPS elicited the activation of MAPKs (ERK, p38 MAPK, JNK) and Akt. However, the addition of VU0155069 did not inhibit the activation of these kinases by LPS in BMDMs (Fig. 4A). The activation of STAT3, IκB degradation and nuclear translocation of NF-κB (markers for NF-κB activation) by LPS stimulation were also not inhibited by VU0155069 (Fig. 4A,B). One final outcome of signal 1 induced by LPS in macrophages is the upregulation of NLRP3 expression^20^. However, addition of VU0155069 did not inhibit the increased NLRP3 expression induced by LPS stimulation in BMDMs (Fig. 4C). The results suggest that VU0155069 does not inhibit LPS-induced signal 1.

**Figure 5:**
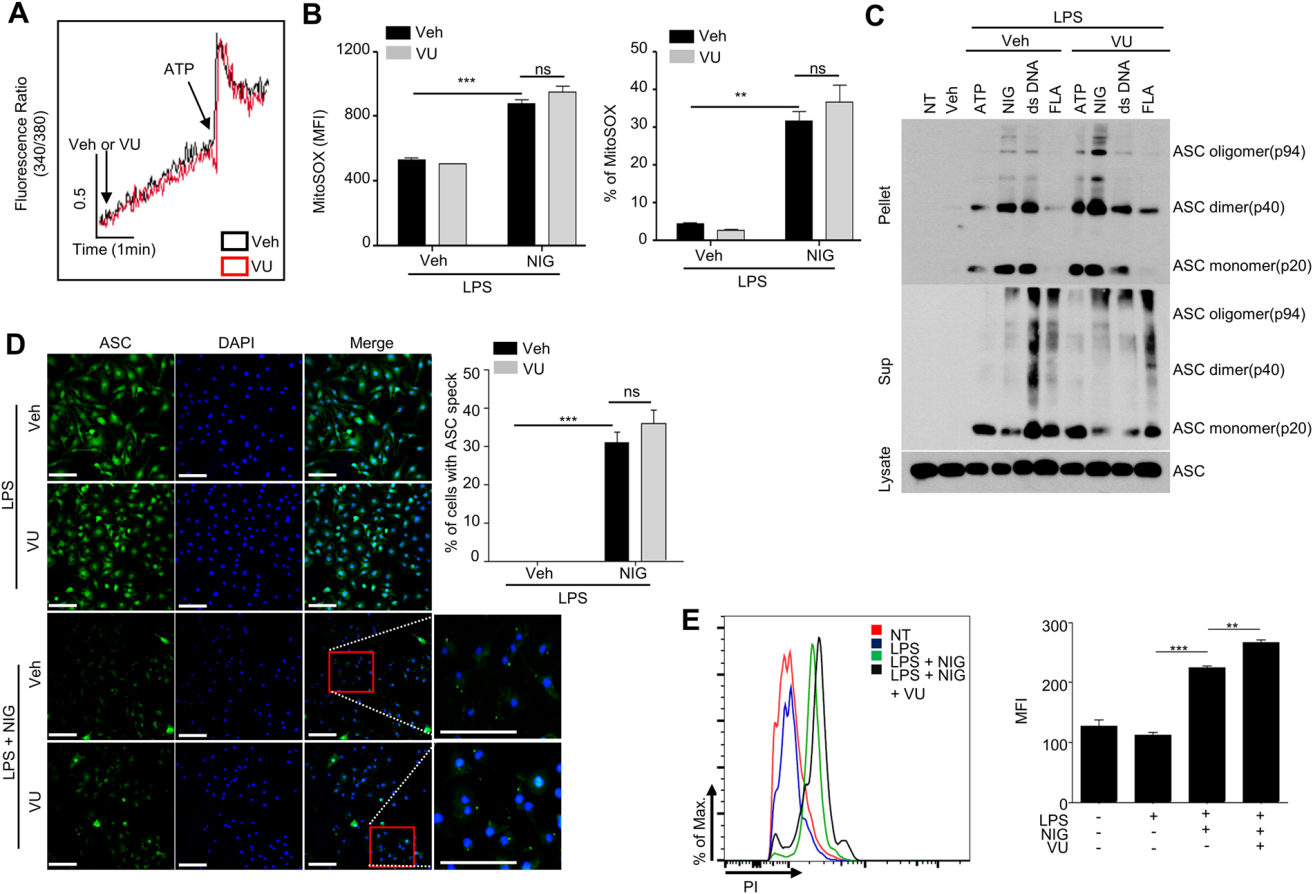
VU0155069 does not affect LPS-stimulated ASC oligomerization by signal 2. (**A**) Fura-2 loaded BMDMs were stimulated with ATP (5 mM) in the presence of vehicle (0.05% DMSO) or VU0155069 (10 μM). Intracellular calcium levels were measured using a spectrofluorophotometer. (**B**) BMDMs were stimulated with LPS (1 μg/ml) for 4 h prior to adding nigericin (NIG) (10 μM) for 30 min in the presence of vehicle (0.05% DMSO) or VU0155069 (10 μM). The levels of mitochondrial ROS were measured by staining the cells with MitoSOX red fluorescent dye with flow cytometry. (**C**) BMDMs were stimulated with LPS (1 μg/ml) for 4 h prior to adding ATP (5 mM), nigericin (NIG) (10 μM) for 30 min, dsDNA (1 μg/ml) or flagellin (FLA) (1 μg/ml) for 16 h in the presence of vehicle (0.05% DMSO) or VU0155069 (10 μM). Different forms of ASC were analyzed by Western blotting from supernatants or cell lysates. Full blots in Supplementary Fig. S3. (**D**) BMDMs were stimulated with LPS (1 μg/ml) for 4 h prior to adding nigericin (NIG) (10 μM) for 30 min in the presence of vehicle (0.05% DMSO) or VU0155069 (10 μM). ASC speck formation was measured by immunofluorescent staining using anti-ASC antibody. Images were obtained by confocal microscopy. (**E**) BMDMs were stimulated with LPS (1 μg/ml) for 4 h prior to adding nigericin (NIG) (10 μM) and PI (5 μM) for 30 min. The cells were analyzed by Flow cytometry. Mean ± SEM (n = 3 for **B**,**E** right, n = 6~10 for **D** right). The data are representative of two independent experiments (**A,C,D** left, **E** left). Scale bar, 100 μm (**D** left). **p < 0.01, ***p < 0.001 by one-way ANOVA followed by Tukey’s multiple-comparisons test. ns, not significant.

Previous reports demonstrated that K^+^ efflux and mitochondrial ROS generation are required for proper activation of the NLRP3 inflammasome complex^21,22^. Although it is controversial whether calcium influx is essential for the activation of NLRP3 inflammasomes by ATP^21^, calcium influx is also observed during the activation of NLRP3 inflammasomes by ATP. Here, we investigated if VU0155069 affect calcium influx caused by ATP, an important stimulus of signal 2. As shown in Fig. 5A, VU0155069 did not affect ATP-stimulated calcium influx in BMDMs. VU0155069 also failed to affect mitochondrial ROS generation caused by LPS + nigericin in BMDMs (Fig. 5B). ASC oligomerization is accompanied by NLRP3 inflammasome activation^23^. In this study, we also observed that stimulation of BMDMs with four different combinations of stimuli (LPS + ATP, LPS + nigericin, LPS + dsDNA, LPS + flagellin) elicited ASC oligomerization, which was detected by Western blot analysis using anti-ASC antibody. Addition of VU0155069 did not inhibit ASC oligomerization induced by the four different combinations of stimuli (Fig. 5C). Immuno-fluorescence staining experiments showed that stimulation of BMDMs with LPS + nigericin caused formation of ASC specks, which was not affected by VU0155069 (Fig. 5D). The results suggest that VU0155069 does not inhibit ASC oligomerization in response to LPS plus nigericin in BMDMs.

A previous report demonstrated that a pore-forming protein gasdermin regulates IL-1β secretion from living macrophages^24^. Since VU0155069 blocked IL-1β secretion caused by LPS + nigericin in BMDMs, we examined whether VU0155069 block gasdermin-dependent pore formation in BMDMs by PI staining. We found that stimulation of BMDMs with LPS + nigericin significantly increased PI^+^ cells, suggesting gasdermin D is cleaved and pores are formed. Addition of VU0155069 did not decrease PI^+^ cells caused by LPS + nigericin (Fig. 5E). The results suggest that VU0155069-induced inhibitory effects on IL-1β secretion is not mediated by blocking gasdermin-dependent pore formation.

### VU0155069 indirectly inhibits caspase-1 activity

**Figure 6:**
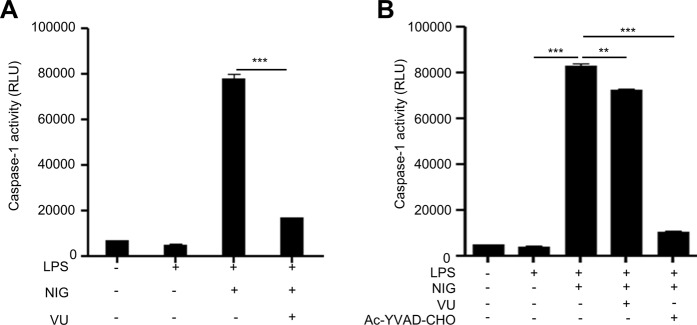
VU0155069 indirectly inhibits caspase-1 activity in BMDMs. (**A**) BMDMs were stimulated with LPS (1 μg/ml) for 4 h prior to adding nigericin (NIG) (10 μM) for 30 min in the absence or presence of VU0155069 (10 μM). (**B**) BMDMs were stimulated with LPS (1 μg/ml) for 4 h prior to adding nigericin (NIG) (10 μM) for 30 min. After harvesting supernatants, VU0155069 (10 μM) or caspase-1 inhibitor (Ac-YVAD-CHO) (1 μM) was added to the supernatants. Caspase-1 activity was measured from the supernatants using a caspase-1 assay kit (**A,B**). Mean ± SEM (n = 4 for **A,B**). **p < 0.01, ***p < 0.001 by one-way ANOVA followed by Tukey’s multiple-comparisons test.

Next, we examined if VU0155069 affects caspase-1 activity. Stimulation of BMDMs with LPS + nigericin strongly increased caspase-1 activity. Addition of VU0155069 prior to the stimuli (LPS + nigericin) almost completely blocked caspase-1 activity (Fig. 6A). To further test if VU0155069 directly affects caspase-1 activity, we added VU0155069 to the supernatants harvested from LPS + nigericin-stimulated BMDMs. VU0155069 only slightly inhibited caspase-1 activity when added to the supernatants (Fig. 6B). As a positive control, we observed that addition of caspase-1 inhibitor to the BMDM supernatants stimulated by LPS + nigericin strongly inhibited caspase-1 activity (Fig. 6B). The results suggest that VU0155069 inhibit caspase-1 activity indirectly rather than directly.

### PLD1 is not involved in inflammasome activation in BMDMs

**Figure 7:**
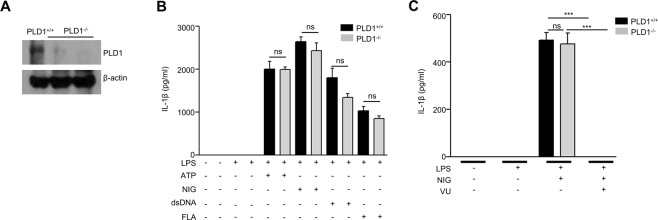
PLD1 is not involved in inflammasome activation in BMDMs. (**A**) Western blot of PLD1 from BMDMs generated from PLD1^+/+^ or PLD1^−/−^ mice. Full blots in Supplementary Fig. S4. (**B**) BMDMs generated from PLD1^+/+^ or PLD1^−/−^ mice were stimulated with LPS (1 μg/ml) for 4 h, and then with ATP (5 mM) for 30 min, nigericin (NIG) (10 μM) for 30 min, dsDNA (1 μg/ml) or flagellin (FLA) (1 μg/ml) for 16 h. (**C**) BMDMs generated from PLD1^+/+^ or PLD1^−/−^ mice were stimulated with LPS (1 μg/ml) for 4 h, and then with nigericin (NIG) (10 μM) for 30 min in the absence or presence of VU0155069 (10 μM). The levels of IL-1β were measured from supernatants using ELISA. Mean ± SEM (n = 3 for **B,C**). ***p < 0.001 by one-way ANOVA followed by Tukey’s multiple-comparisons test. ns, not significant.

Our finding on the inhibitory effects of VU0155069 on inflammasome activation and subsequent IL-1β production in macrophages led us to examine the role of PLD1 in the process in BMDMs. At first, we verified that BMDMs generated from PLD1^−/−^ mice do not express PLD1 (Fig. 7A). BMDMs generated from PLD1^+/+^ or PLD1^−/−^ mice were stimulated with several inflammasome-activating signals (LPS + ATP, LPS + nigericin, LPS + dsDNA, LPS + flagellin), and secreted IL-1β levels were measured. Unexpectedly, PLD1 deficiency did not affect IL-1β production induced by these inflammasome-activating signals (Fig. 7B). Moreover, VU0155069 still almost completely inhibited LPS + nigericin-induced IL-1β production in BMDMs generated from PLD1^−/−^ mice (Fig. 7C). The results suggest that the PLD1 protein itself is not involved in the regulation of inflammasome activation, and VU0155069-induced inhibitory effects on inflammasome activation is mediated in a PLD1-independent manner.

## Discussion

In this study we found that administration of VU0155069, an inhibitor of PLD1, effectively induces therapeutic effects against experimental polymicrobial sepsis. The therapeutic effects of VU0155069 on sepsis are associated with decreased production of IL-1β, and VU0155069 strongly inhibited inflammasome activation and subsequent IL-1β production from BMDMs. On the mode of action of VU0155069 involved in inhibition of the inflammasome, we found that VU0155069 did not inhibit LPS-stimulated signal 1 or ASC oligomerization. However, VU0155069 effectively inhibited caspase-1 activity in response to LPS + nigericin, in an indirect manner. Based on the results obtained from this study, we suggest that VU0155069 is a novel regulator of the inflammasome and inflammatory cytokine IL-1β production.

Unlike other inflammatory cytokines such as TNF-α and IL-6, IL-1β production is regulated by using specialized machinery in form of the inflammasome, that requires at least two different signals (signal 1 and signal 2)^20^. Here, we observed that a PLD1 inhibitor VU0155069 strongly blocked inflammasome activation, IL-1β production, and pyroptosis from BMDMs (Figs 2 and 3). Through several experiments on the molecular mechanism involved in inhibition of the inflammasome by VU0155069, we observed that VU0155069 did not affect LPS-induced signal 1 (the activation of MAPK, Akt, and NF-κB), signal 2 caused by ATP (calcium influx), or ASC oligomerization and speck formation (Figs 4 and 5). However, VU0155069 strongly inhibited caspase-1 activity elicited by an inflammasome activating signal (LPS + nigericin) in BMDMs (Fig. 6A). In a separate experiment, we found that VU0155069 only slightly inhibited caspase-1 activity *in vitro* (Fig. 6B). VU0155069 inhibited caspase-1 activation and subsequent IL-1β production in response to not only NLRP3 but also AIM2 and NLRC4 (Fig. 2C,F). Although AIM2 and NLRC4 are distinct inflammasome to NLRP3, they are all controlled by caspase-1 activity^5^. Our results suggest that VU0155069 regulates the activity of inflammasome by indirect controlling caspasee-1, and VU0155069 can be used to inhibit these three types of inflammasome. Collectively, our results suggest that VU0155069 may act downstream of ASC oligomerization to induce inhibitory effects on the inflammasome, but not directly on caspase-1. Our findings on the action mode of VU0155069 additionally suggest that an unrevealed regulatory mechanism leading to caspase-1 activation may exist downstream of ASC oligomerization.

Although VU0155069 has been reported to specifically inhibit PLD1 in a previous report^15^ and VU0155069 strongly inhibited inflammasome activation in BMDMs, we observed that PLD1 deficiency in BMDMs did not affect inflammasome activation (Fig. 7B). Moreover, inflammasome-dependent IL-1β production was also almost completely inhibited by VU0155069 from PLD1-deficient BMDMs (Fig. 7C). Our findings suggest that VU0155069 may have a different target from PLD1, that plays a role in regulation of the inflammasome. Since regulation of inflammasome activation is important in the regulation of diverse inflammatory disorders and VU0155069 shows strong inhibitory effects on inflammasome activation independent of PLD1, identification of the target molecule of VU0155069 should be important.

The inflammasome and inflammasome-activated IL-1β production are associated with diverse diseases such as Crohn’s disease and gout^5^. Since we found that VU0155069 strongly inhibited IL-1β production induced by inflammasome-inducing signals by inhibiting caspase-1 activity, VU0155069 can be used to control inflammasome-associated diseases. In this study, we also observed that VU0155069 administration strongly induced therapeutic effects against polymicrobial sepsis, whose pathological progress is also mediated by increased IL-1β. Since we found that VU0155069 showed inhibitory effects on activation of the inflammasome with an unrevealed molecular mechanism to block caspase-1 activation, future studies on the mode of action of VU0155069 would provide novel insights to control inflammasome and inflammasome-related diseases. Thus, VU0155069 can be regarded as a new candidate to control inflammasome-associated disorders.

## Methods

### Mice and CLP model

C57BL/6 mice were purchased from Orient Bio (Seongnam, Korea). PLD1^−/−^ mice were purchased from the Jackson Laboratory (Bar Harbor, ME, USA). All animal experiments were performed in accordance with the guidelines of the Korean Food and Drug Administration. All experiments involving animals received the approval of the Institutional Review Committee for Animal Care and Use at Sungkyunkwan University (Suwon, Korea). The polymicrobial CLP sepsis model was conducted as described previously^14^. Briefly, mice were anesthetized with isoflurane, the cecum was exposed from an abdominal midline incision, ligated and punctured with a 23-gauge needle, and the abdomen sutured. The vehicle (0.5% Tween 80 in PBS) or VU0155069 (Cayman, Ann Arbor, MI, USA) was subcutaneously injected four times into CLP mice 2, 14, 26, and 38 h after CLP.

### Lung histology and TUNEL assay

Vehicle or VU0155069-injected CLP mice were sacrificed at 24 h after surgery. Their lungs were fixed in neutral buffered formalin, sectioned with paraffin, and stained with hematoxylin and eosin for medical diagnosis. Isolated spleens and thymus were used to make frozen sections for the TUNEL assay (Roche, Basel, Switzerland). The sections were placed in Triton X-100 at 4 °C for 2 min for permeation and incubated with TUNEL reagent at 37 °C for 60 min. The percentage of TUNEL-positive cells was counted under a light microscope.

### Quantification of pulmonary edema

Vehicle or VU0155069-injected CLP mice were sacrificed at 24 h after CLP. Whole lungs were harvested and then weighed. Afterwards, the lungs were placed in a 60 °C oven for 48 h. The dry lungs were weighed, and the wet/dry weight ratio was calculated.

### Measurement of CFUs in CLP model

Vehicle or VU0155069-injected CLP mice were sacrificed at 24 h after surgery. Peritoneal fluid was harvested and cultured overnight on Trypticase soy agar plates at 37 °C incubator. CFUs were determined as described previously^14^.

### Generation of mouse BMDMs

Mouse BMDMs were generated according to a previous report^25^. Briefly, femur and tibia were obtained from 5 week old mice. After flushing the bone marrow cells from the femur and tibia, red blood cells were lysed using ACK lysis buffer (Gibco, Waltham, MA, USA). The bone marrow cells were cultured in α-MEM containing 10% FBS for one day, and non-adherent cells were further cultured for three days with 30 ng/ml M-CSF (Peprotech, Rocky Hill, NJ, USA).

### Measurement of cytokines and LDH

Peritoneal fluid was harvested from vehicle or VU0155069-injected CLP mice 24 h after injection. Cytokine (TNF-α, IL-6, IL-1β, IL-22, IFN-γ) levels were measured by ELISA (eBioscience, San Diego, CA, USA). To measure the levels of IL-1β and TNF-α, isolated BMDMs were stimulated with LPS (1 μg/ml) for 4 h, followed by treatment with an inflammasome inducer (nigericin or monosodium urate, dsDNA, flagellin). LDH activity was measured at 490 nm in conditional media from inflammasome-induced BMDMs using the LDH assay kit (Promega, Madison, WI, USA).

### Western blot analysis

BMDMs were cultured in inflammasome-inducing conditions (LPS + nigericin, LPS + dsDNA, or LPS + flagellin). Cultured cells and conditioned media were harvested, and the cells lysed with lysis buffer (20 mM HEPES [pH 7.2], 10% glycerol, 150 mM NaCl, 1% Triton X-100, 50 mM NaF, 1 mM Na_3_VO_4_, 10 μg/ml leupeptin, 10 μg/ml aprotinin, and 1 mM PMSF). Conditioned media were applied to the methanol/chloroform protein precipitation. Protein extracts were separated by SDS-PAGE, transferred to a polyvinylidene fluoride membrane and subjected to Western blot analysis. The level of specific proteins was detected with antigen-antibody complexes using enhanced chemiluminescent substrate and horseradish peroxidase activity. Antibodies used in Western blot analysis are anti-pERK, anti-pp38, anti-pJNK, anti-pAkt, anti-β-actin, anti-α-Tubulin, anti-PLD1 and anti-pSTAT3 (Cell Signaling Technology, Danvers, MA, USA), anti-caspase-1 and anti-NLRP3 (Adipogen, San Diego, CA, USA), anti-IL-1β (R&D, Minneapolis, MN, USA), and anti-p65, anti-lamin B and anti-ASC (Santa Cruz Biotechnology, Dallas, Texas, USA).

### Measurement of cytosolic calcium levels

Isolated mouse BMDMs were incubated with Fura-2/AM (3 μM) at 37 °C for 50 min. Fura-2/AM loaded BMDMs in Locke’s solution (154 mM NaCl, 5.6 mM KCl, 1.2 mM MgCl_2_, 5 mM HEPES, pH 7.3, 10 mM glucose, 2.2 mM CaCl_2_, and 0.2 mM EGTA) were stimulated with ATP in the presence of vehicle or VU0155069, and changes in fluorescence ratios were monitored using a spectrofluorophotometer (SHIMADZU, Kyoto, Japan).

### Measurement of mitochondrial ROS

BMDMs were stimulated with LPS + nigericin in the present of vehicle or VU0155069. Then the cells were treated with 5 μM MitoSOX reagent (Sigma, St. Louis, MO, USA), and further incubated at 37 °C for 20 min. The levels of mitochondrial ROS were measured with flow cytometry.

### Measurement of ASC oligomerization

Culture supernatants and cell pellets were collected from inflammasome-inducing BMDMs. Collected samples were lysed in lysis buffer (20 mM HEPES [pH 7.2], 10% glycerol, 150 mM NaCl, 1% Triton X-100, 50 mM NaF, 1 mM Na_3_VO_4_, 10 μg/ml leupeptin, 10 μg/ml aprotinin, and 1 mM PMSF). The cell lysate was centrifuged, and the pellets resuspended in PBS. Resuspended pellets were treated with disuccinimidyl suberate (DSS, 2 mM) for 30 min. Then the pellets were resuspended in 2X sample buffer and separated on 12% SDS-PAGE. ASCs were detected by Western blot analysis using anti-ASC antibodies.

### Confocal microscopy

Poly-L-lysin was coated on a slide glass overnight, and isolated BMDMs were placed on the slide. After washing with Opti MEM, 1 μg/ml of LPS was added for 4 h, and subsequently nigericin was added for 1 h, and then the samples were fixed with 4% paraformaldehyde for 20 min at room temperature. Next, permeabilization with 0.2% Triton X-100 was performed. After blocking for 1 h with 1% BSA in PBS, samples were stained with ASC antibody for 3 h and then stained for 1 h with anti-mouse Alexa Fluor 488. After staining the nuclei with Hoechst for 5 min, samples were observed with confocal microscopy, and photo images were analyzed with ZEN software.

### Measurement of membrane pore formation

Isolated BMDMs were stimulated with LPS (1 μg/ml) for 4 h, followed by treatment with nigericin (10 μM) and Propidium iodide (5 μM) for 30 min. The stimulated cell was centrifuged at 1500 rpm for 5 min. PI^+^ cells were detected by flow cytometry (BD FACSCanto™ II).

### Measurement of caspase-1 activity

Isolated BMDMs were stimulated with LPS (1 μg/ml) for 4 h, followed by treatment with nigericin (10 μM) for 30 min. Supernatant was harvested from inflammasome induced BMDMs to measure caspase-1 activity. Z-WEHD substrate was added in supernatant on 96 well plate using caspase Glo 1 inflammasome assay kit (Promega, Madison, WI, USA) for 1 h. Caspase-1 activity was measured using a GLOMAX luminometer (Promega, Madison, WI, USA).

### Statistical analysis

Results were evaluated using GraphPad Prism software. Statistical analysis was performed using ANOVA or t-test. Survival data analysis was performed by log-rank test. All results are expressed as the mean ± SEM. A *p* value < 0.05 was considered statistically significant.

## Supplementary information

Supplementary Figures
