# Supplementary material for: VU0155069 inhibits inflammasome activation independent of phospholipase D1 activity
Source: Sci Rep. 2019 Oct 4;9:14349. doi: 10.1038/s41598-019-50806-9 (PMC6778193; doi:10.1038/s41598-019-50806-9)
Supplement: Supplementary file 1 — Supplementary Figures [file 41598_2019_50806_MOESM1_ESM.pdf]

VU0155069 inhibits inflammasome activation independent of phospholipase D1 activity

Sung Kyun Lee,[1,3,4] Ye Seon Kim,[1,3] Geon Ho Bae, [1] Ha Young Lee,[1] and Yoe-Sik Bae [1,2,*]

[1]Department of Biological Sciences, Sungkyunkwan University, Suwon 16419, Republic of Korea.

[2]Department of Health Sciences and Technology, SAIHST, Sungkyunkwan University, Seoul 06351, Republic of Korea.

Running title: A new inflammasome inhibitor

[3]These authors equally contributed to this work.

[*]Corresponding author: Tel: 82-31-290-5914; Fax: 82-31-290-7015;
E-mail: yoesik@skku.edu (Y.B.)

[4]Present address: Institute for Stem Cell & Regenerative Medicine Research of Albert Einstein College of Medicine, Bronx, NY 10461, USA

Keywords: inflammasome, VU0155069, macrophages, interleukin 1β, caspase-1

Supplementary Figures

Supplementary Figure S1

Fig. 2E

Fig. 2F

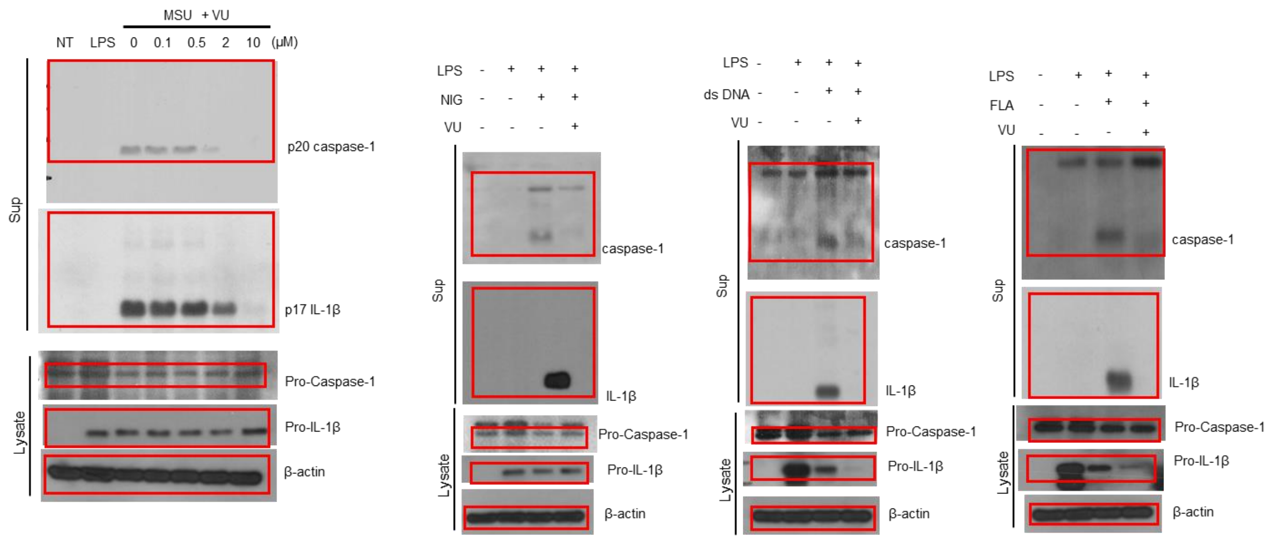

Supplementary Figure S1: Full length blots of Figures 2E and 2F. Red lines show the cropping locations.

Supplementary Figure S2

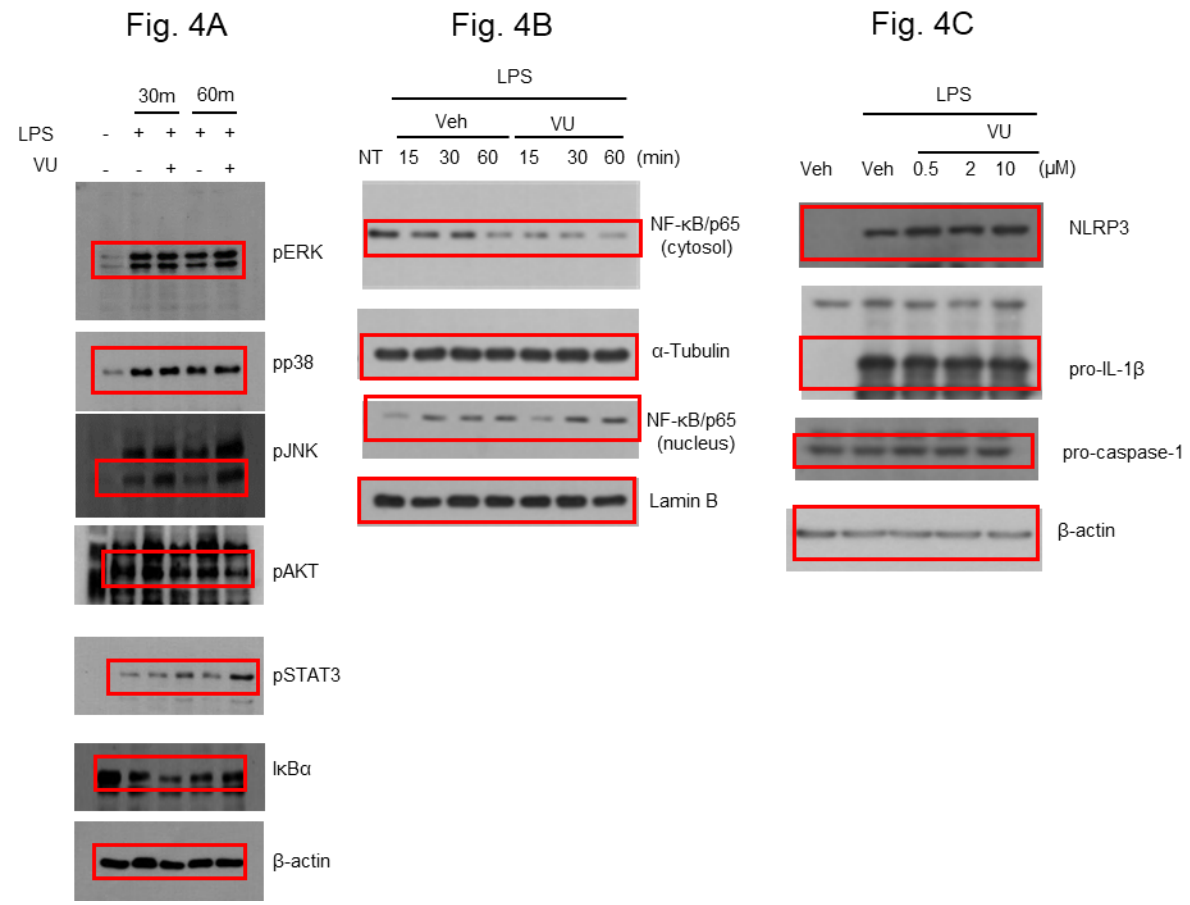

Supplementary Figure S2: Full length blots of Figures 4A, 4B and 4C. Red lines show the cropping locations

Supplementary Figure S3

Fig. 5C

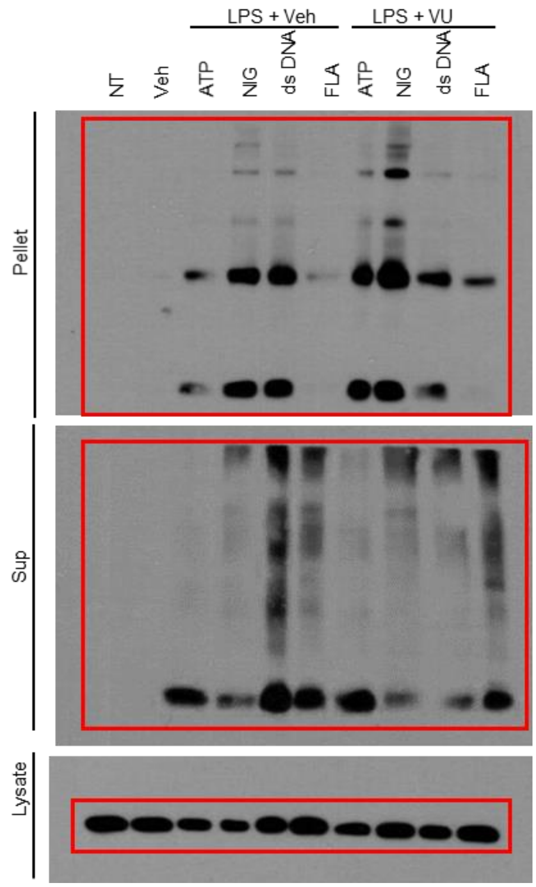

Supplementary Figure S3: Full length blots of Figure 5C. Red lines show the cropping locations.

Supplementary Figure S4

Fig. 7A

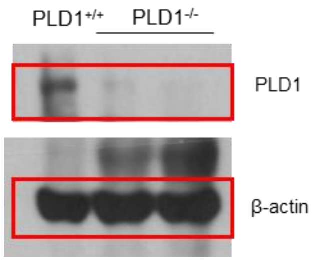

Supplementary Figure S4: Full length blots of Figure 7A. Red lines show the cropping locations.
